# Pathogenicity of Seneca Valley virus in pigs and detection in *Culicoides* from an infected pig farm

**DOI:** 10.1186/s12985-021-01679-w

**Published:** 2021-10-21

**Authors:** Jinyong Zhang, Chenghui Li, Yuan Meng, Yubiao Xie, Ning Shi, He Zhang, Chengdong Yu, Fulong Nan, Changzhan Xie, Zhuo Ha, Jicheng Han, Zhuoxin Li, Qiuxuan Li, Peng Wang, Xu Gao, Ningyi Jin, Huijun Lu

**Affiliations:** 1 Changchun Veterinary Research Institute, Chinese Academy of Agricultural Sciences, 666 Liuying West Road, Changchun, 130122 People’s Republic of China; 2 College of Animal Science and Technology, Jilin Agricultural University, Changchun, 130118 People’s Republic of China; 3 College of Agricultural, Yanbian University, 977 Gongyuan Road, Yanji, 133002 People’s Republic of China; 4 College of Veterinary Medicine, Jilin University, Changchun, 130012 People’s Republic of China; 5 Jiangsu Co-Innovation Center for the Prevention and Control of Important Animal Infectious Disease and Zoonoses, Yangzhou University, Yangzhou, 225009 People’s Republic of China

**Keywords:** Seneca Valley virus (SVV), Pathogenicity, Pig, *Culicoides*

## Abstract

**Background:**

Porcine vesicular disease is caused by the Seneca Valley virus (SVV), it is a novel *Picornaviridae,* which is prevalent in several countries. However, the pathogenicity of SVV on 5–6 week old pigs and the transmission routes of SVV remain unknown.

**Methods:**

This research mainly focuses on the pathogenicity of the CH-GX-01-2019 strain and the possible vector of SVV. In this study, 5–6 week old pigs infected with SVV (CH-GX-01-2019) and its clinical symptoms (including rectal temperatures and other clinical symptoms) were monitored, qRT-PCR were used to detect the viremia and virus distribution. Neutralization antibody assay was set up during this research. Mosquitoes and *Culicoides* were collected from pigsties after pigs challenge with SVV, and SVV detection within mosquitoes and *Culicoides* was done via RT-PCR.

**Results:**

The challenged pigs presented with low fevers and mild lethargy on 5–8 days post infection. The viremia lasted more than 14 days. SVV was detected in almost all tissues on the 14th day following the challenge, and it was significantly higher in the hoofs (vesicles) and lymph nodes in comparison with other tissues. Neutralizing antibodies were also detected and could persist for more than 28 days, in addition neutralizing antibody titers ranged from 1:128 to 1:512. Mosquitoes and *Culicoides* were collected from the pigsty environments following SVV infection. Although SVV was not detected in the mosquitoes, it was present in the *Culicoides*, however SVV could not be isolated from the positive *Culicoides*.

**Conclusions:**

Our work has enriched the knowledge relating to SVV pathogenicity and possible transmission routes, which may lay the foundation for further research into the prevention and control of this virus.

**Supplementary Information:**

The online version contains supplementary material available at 10.1186/s12985-021-01679-w.

## Background

Seneca Valley virus (SVV) is a single-stranded positive-sense RNA virus, which belongs to genus Senecavirus within the family *Picornaviridae* [1, 2]. The SVV genome is approximately 7.3 kb long and contains an open reading frame (ORF) encoding one polyprotein (2181 Amino Acids), encoding 12 mature proteins (L-VP4-VP2-VP3-VP1-2A-2B-2C-3A-3B-3C-3D) [3, 4]. SVV-001 was the first SVV strain to be identified and isolated, having been reported as a cell contaminant in USA in 2002, used as a drug to treat cancer [3, 5]. In 2007, porcine infection with SVV was confirmed in Canada, followed by infection cases reported from America, Brazil, China, Columbia, Thailand and Vietnam [6–12]. The main clinical symptoms of SVV infection are blisters on the snout and/or hooves, which made it difficult to distinguish from food-and-mouth disease virus (FMDV), vesicular stomatitis virus (VSV), swine vesicular disease virus (SVDV), and vesicular exanthema of swine virus (VESV) [2, 12].

In China, porcine infection with SVV was first reported in Guangdong Province in 2015 [9]. Following this discovery small-scale prevalence of SVV cases in Hubei [13], Fujian, Henan [14], Heilongjiang [15], Guangxi [16], Hebei [17], Anhui [18], Shandong [19], Sichuan [20] and Gansu Province [17] were reported. In addition, SVV-positive events were reported in Shanghai, Liaoning, Xinjiang, Guizhou, Yunnan, Hainan Province, but no SVV strains were isolated from the infected pigs [16, 17]. Presently, multiple types of SVV strains circulate within the pig breeding environment in China, and the virulence of each strain differs. Up until now, the transmission routes of SVV and its infected population have not been elucidated. In this paper, the pathogenicity of CH-GX-01-2019 in pigs was studied, environmental samples (mosquitoes and *Culicoides*) from the pigsties containing pathogenic pigs were analyzed, and the possible transmission routes of SVV have been speculated.

## Materials and methods

### Viruses and cells

The SVV strain CH-GX-01-2019 (GenBank accession number: MT457474), was isolated and preserved within the laboratory. Baby hamster kidney (BHK-21) cell line was maintained in the Dulbecco’s modified Eagle’s medium (DMEM; Hyclone, USA) and supplemented with 5% fetal bovine serum (FBS; Hyclone, USA), 1% penicillin (10,000 units/ml)-streptomycin (10,000 units/ml) solution (Hyclone, USA) and cultured at 37 °C under 5% CO_2_ conditions, from which SVV was propagated and titrated.

### Animal inoculation

10 pigs (5–6 week pigs) were randomly allocated into 2 experimental groups (5 pigs in each group)—a PBS-inoculated group and a SVV-inoculated group. SVV was negative in terms of serology and etiology, and was detected using a neutralizing antibody and also by reverse transcription polymerase chain reaction (RT-PCR) [16, 21]. Pigs were challenged with either 10 ml PBS or 10 ml SVV (10^7.5^TCID_50_/ml), via administration of 5 ml into each nostril.

### Clinical symptom analysis

Throughout the study, several clinical symptoms were monitored. The rectal temperatures of each pig were measured for 22 consecutive days. Clinical symptoms including mental state, food intake, vesicular lesions, lameness and other symptoms were also observed and recorded.

### Viremia and viral load detection of SVV

Blood samples were collected for virological examination on day 3, 7, 10, 14, 21, and 28 post-infection. Total RNA extractions were performed on each sample (52 samples in total) according to the manufacturer’s instructions using RNA TRIzol reagent (Sangon, China). Using random primers (primer 9) and M-MLV reverse transcriptase (Takara, China), cDNA was obtained according to the manufacturer’s instructions. SYBR Green I quantitative real-time PCR (qRT-PCR) was used to detect the SVV viral load. The detection primers were designed using Primer Premier 5 (Additional file 1: Table S1).

Two pigs in the PBS-inoculated group and two pigs with obvious clinical symptoms in the SVV-inoculated group were euthanized at 14 days post infection (dpi), pigs from each group underwent humane killing and anatomy. The heart, liver, spleen, lung, kidney, submaxillary lymph nodes (submaxillary LN), inguinal lymph nodes (inguinal LN), intestine, tongue, tonsil and hoof (with blister) were collected from each animal in order to determine virus distribution. Total cDNA extraction and qRT-PCR were performed as described above.

### Virus neutralization assay

Serum samples were collected at 0, 7, 10,14, 21, and 28 dpi, and neutralizing antibody titers in the serum from pigs challenged with CH-GX-01-2019 were detected using a virus neutralizing antibody test (VNT), as described previously [21, 22]. The serum samples were inactivated at 56 ℃ for 30 min, then serial twofold dilutions (1:4 to 1:4096) were diluted with DMEM into 96-well plates. The diluted serum samples (50 µl) were then incubated with 200 TCID_50_ CH-GX-01-2019 (50 µl) and then incubated at 37 °C for 1 h. The BHK-21 cells (100 µl) were added to each well (at a concentration of approximately 10,000 cells per well), and the plates were further incubated at 37 °C for 72 h. Neutralizing antibody titers were determined via observation of the cytopathic effect (CPE) in the BHK-21 cells.

### Sample collection and detection of mosquitoes and Culicoides

A mosquito trap was set about 10 m away from the pigsty to catch *Culicoides* and mosquitoes. The mosquitoes and *Culicoides* were collected for 14 days (1–14 dpi). About 1000 mosquitoes and 54,000 *Culicoides* were collected. The mosquitoes were randomly divided into 10 groups with 100 individuals per group and the *Culicoides* were randomly divided into 50 groups, each containing 1000 individuals. The 50 samples were combined into 10 samples for detection purposes, and then the positive samples were selected for RT-PCR analysis as described in the supplementary conditions and results). Total cDNA was extracted as previously described with primers designed using Primer Premier 5 (Additional file 1: Table S2). Sequencing and alignment of the full genomes were performed according to previous methods [16]. Using the BHK-21 cell line, SVV was isolated as previously described and the isolated SVV was identified using RT-PCR and an indirect immunofluorescence assay (IFA) [10, 16].

### Statistics

Data were analyzed using GraphPad Prism (version 6.0) software (GraphPad Software Inc., La Jolla, CA). All data are presented as the mean ± SD. Differences in levels of average daily gain, viral load detection in tissues and neutralizing antibody titers between different groups were determined by one-way repeated measures ANOVA and least significance difference (LSD). Differences were considered statistically significant when *P* < 0.05 (* indicates *P* < 0.05).

## Results

### Clinical symptoms of infection

**Fig. 1:**
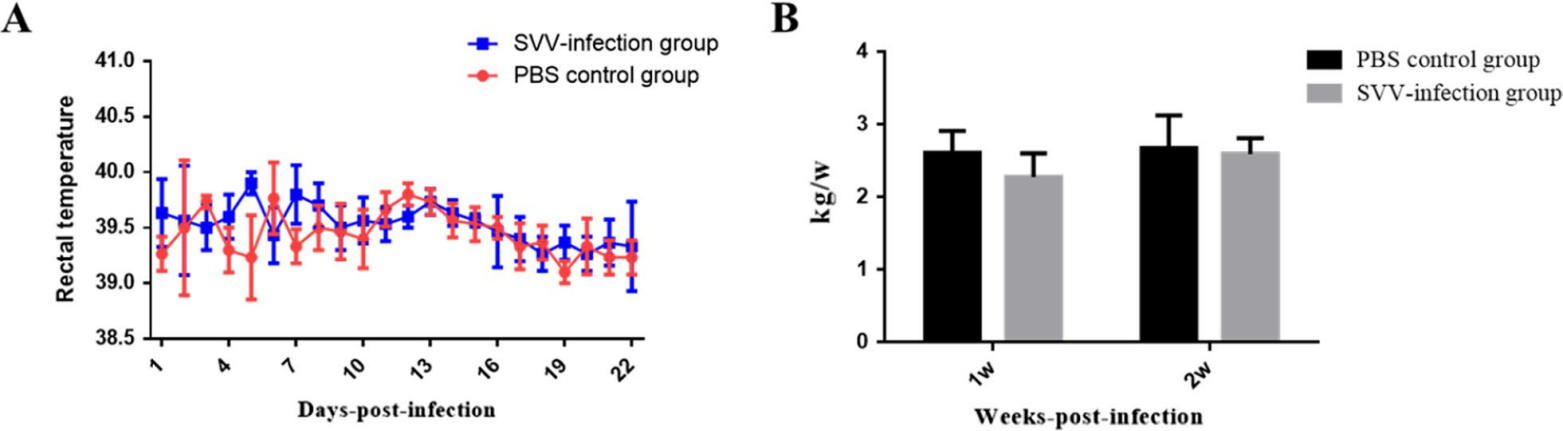
Evaluation of clinical symptoms. The rectal temperature of SVV-infection group was higher than PBS control group (**A**). Average weekly weight gain of the SVV-infection group showed no significant differences compared to the PBS control group (**B**)

**Fig. 2:**
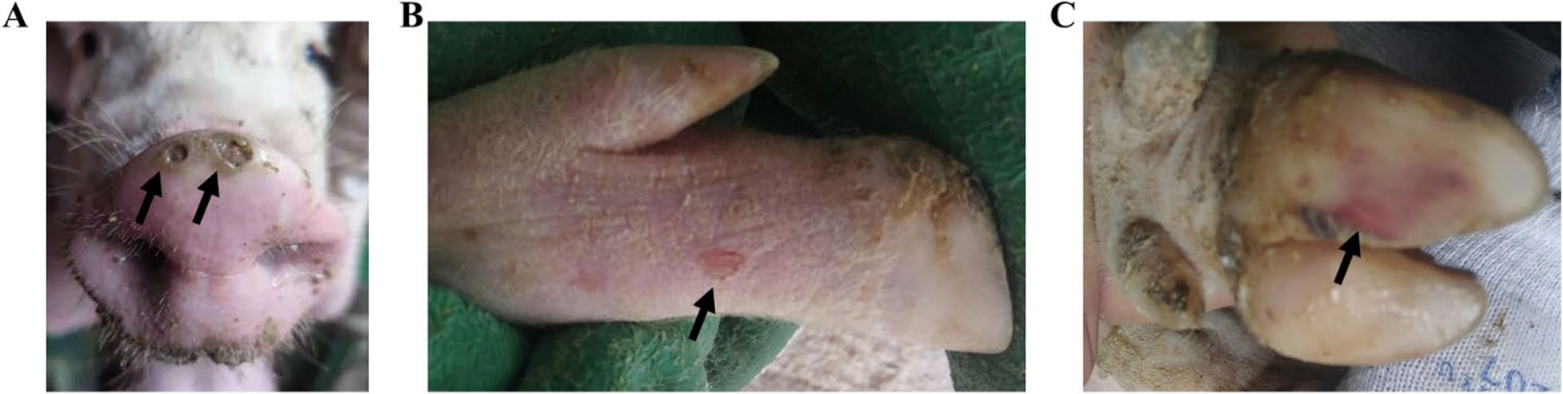
Vesicular lesion observed on pigs infected with SVV. Lesions was observed on snout (**A**) and feet (**B**), blood-like lesions on the hoof (**C**)

The rectal temperatures of the SVV-inoculation groups showed transient increases but did not exceed 40 ℃ in the 5–8 dpi period (Fig. 1A). Pigs infected with SVV showed symptoms of mild depression 5–10 dpi, whoever, feed intake and weights showed no significant differences in comparison to the control PBS group (Fig. 1B). From day 5 post-infection, the SVV group showed erythema near the pig hooves which was vesicular (Fig. 2B). Thereafter vesicular lesions were observed on the snout (day 6, Fig. 2A), and fluid-filled vesicles burst in the hooves and blood-like lesions were observed (7–14 dpi, Fig. 2C). In order to further describe the SVV infected clinical symptoms more accurately, the incidence of SVV infection in the pigs was also analyzed (Table 1).

**Table 1:** Statistics of clinical symptoms of the pigs infected with the SVV

Groups	Lesion on the snout		Lesion on the hoof	Blood-like lesions on the hoof
PBS control group	0/5	0/5		0/5
SVV-infection group	1/5	2/5		4/5

### Viremia analysis and viral load detection in tissues

**Fig. 3:**
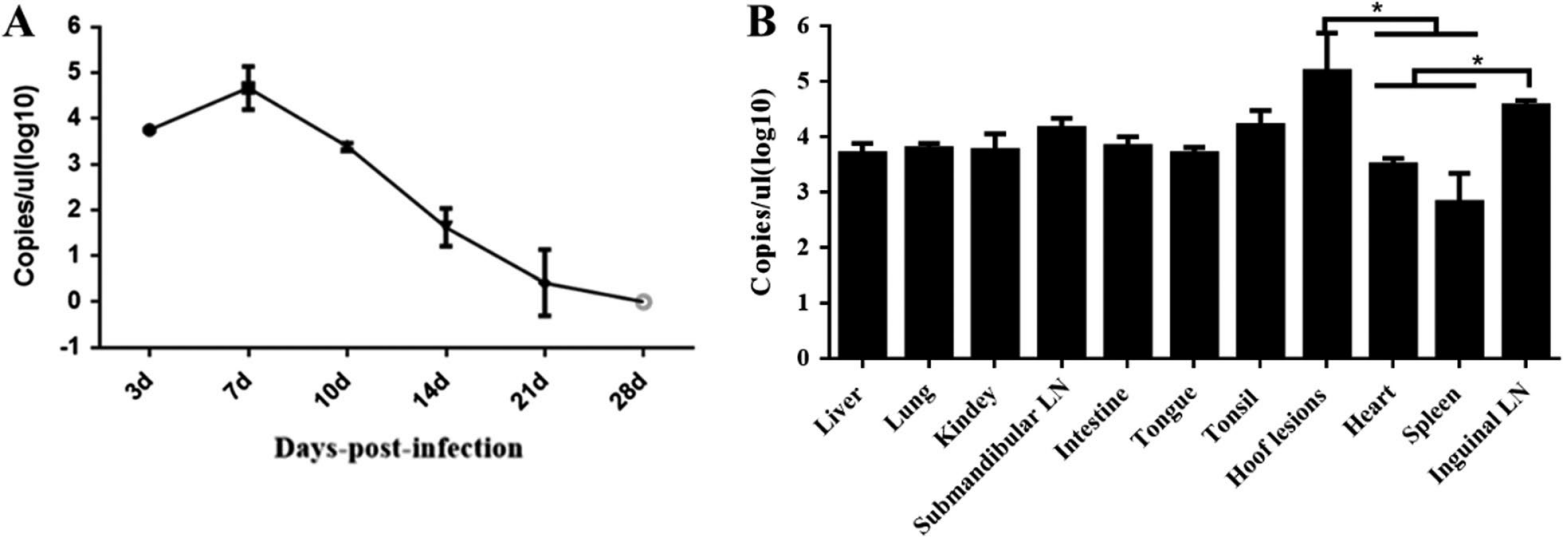
Viremia detection and virus distribution detection. Detection of duration of viremia, and viremia persisted for more than 14 days following infection with SVV in pigs (**A**). Viral load detection in tissues and SVV can be detected in all tissues, viral load in hoof lesions and submaxillary LN were significantly higher than those in heart and spleen (**B**)

In order to analyze the duration of viremia caused by SVV in pigs, blood samples were collected at post-infection days 3, 7, 10, 14, 21, and 28. qRT-PCR was established for viral load detection (Additional file 2: Fig. S1). The results showed that SVV could be detected in the blood on the 3rd day after the challenge, and the viral load peaked at 7 dpi, at about 10^4.5^ copies/μl. Thereafter, the viral load in blood decreased rapidly to about 10^1.5^ copies/μl at 14 dpi, until elimination at 21 dpi. Therefore, in this study, viremia persisted for more than 14 days following infection with SVV in pigs (Fig. 3A).

Pigs from each group underwent humane killing and anatomy at 14 dpi. The tissues the including heart, liver, spleen, lung, kidney, submaxillary LN, inguinal LN, intestine, tongue, tonsil and hooves with blisters were collected and the cDNA was obtained from them for qRT-PCR analysis. The results showed that SVV was detected in all of these tissues, with the viral loads highest in the hooves with blisters (at approximately 10^5^ copies/μl). The virus copies within the submaxillary LN, inguinal LN and tonsil were higher than 10^4^ copies/μl (Fig. 3B).

### Neutralization antibody levels

**Fig. 4:**
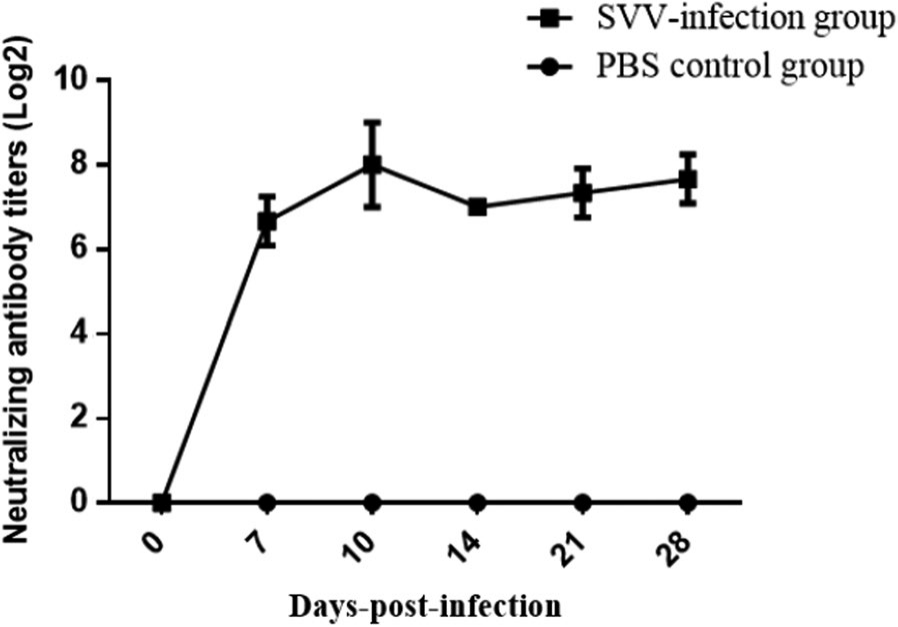
Neutralizing antibody titers of pigs to infection with CH-GX-01-2019 SVV strain. The neutralizing antibody titer curve trend in the graph is plotted for sera from pigs that were not euthanized. Neutralizing antibody titers reached peak at 10 dpi, and neutralizing antibodies persisted for at least 28 days

A neutralization antibody assay was set up during this research to test serum samples collected on 0, 7, 10,14, 21, and 28 dpi. All of the SVV-infected pigs seroconverted and generated neutralizing antibodies on the 7th dpi, and the neutralizing antibodies titers ranged from 1:64 to 1:128. The neutralizing antibody titers reached their highest levels around 10 days post-infection with SVV, and the neutralizing antibody titers reached from 1:128 to 1:512 at that point. The neutralizing antibodies persisted for at least 28 days in the pigs infected with the CH-GX-01-2019 SVV strain, and were largely unabated at around 28 days (Fig. 4 and Table 2).

**Table 2:** Neutralizing antibody titers of pigs to infection with CH-GX-01-2019 SVV strain

Groups	Pig no	Days-post-infection (dpi)					
		0	7	10	14	21	28
PBS control group	1	< 1:4	< 1:4	< 1:4	< 1:4	< 1:4	< 1:4
	2	< 1:4	< 1:4	< 1:4	< 1:4	< 1:4	< 1:4
	3	< 1:4	< 1:4	< 1:4	< 1:4	< 1:4	< 1:4
	4	< 1:4	< 1:4	< 1:4	< 1:4		
	5	< 1:4	< 1:4	< 1:4	< 1:4		
SVV-infection group	6	< 1:4	1:128	1:512	1:128	1:128	1:256
	7	< 1:4	1:64	1:128	1:128	1:256	1:256
	8	< 1:4	1:128	1:256	1:128	1:128	1:128
	9	< 1:4	1:64	1:128	1:128		
	10	< 1:4	1:128	1:128	1:128		

### Detection of SVV in mosquitoes and Culicoides

About 1000 mosquitoes were collected and SVV detection was carried out using RT-PCR. The results showed that SVV was not detected in any of the samples (Additional file 3: Table S3).

About 54,000 *Culicoides* were collected and again SVV infection was detected using RT-PCR. The results showed that four out of ten samples were SVV positive (Additional file 3: Table S3, Fig. S2). When samples 11–30 underwent RT-PCR, 13 out of the 20 samples were SVV positive (Additional file 3: Table S3, Fig. S2). The complete gene sequences of the positive samples were sequenced and spliced successfully, the sequence detected was completely consistent with that shown by the CH-GX-01-2019 strain. Positive sample grinding fluid was to undertake virus isolation from the BHK-21 cells. Blind passages were carried out on the BHK-21 cells for 5 passages, and RT-PCR and indirect immunofluorescence were used for identification purposes for each generation of cells. Cell cultures in passages 3–5 were all SVV negative according to the RT-PCR results. None had visible green fluorescence under indirect immunofluorescence microscopy analysis in any passage. SVV was therefore not isolated from positive *Culicoides*.

**Fig. 5:**
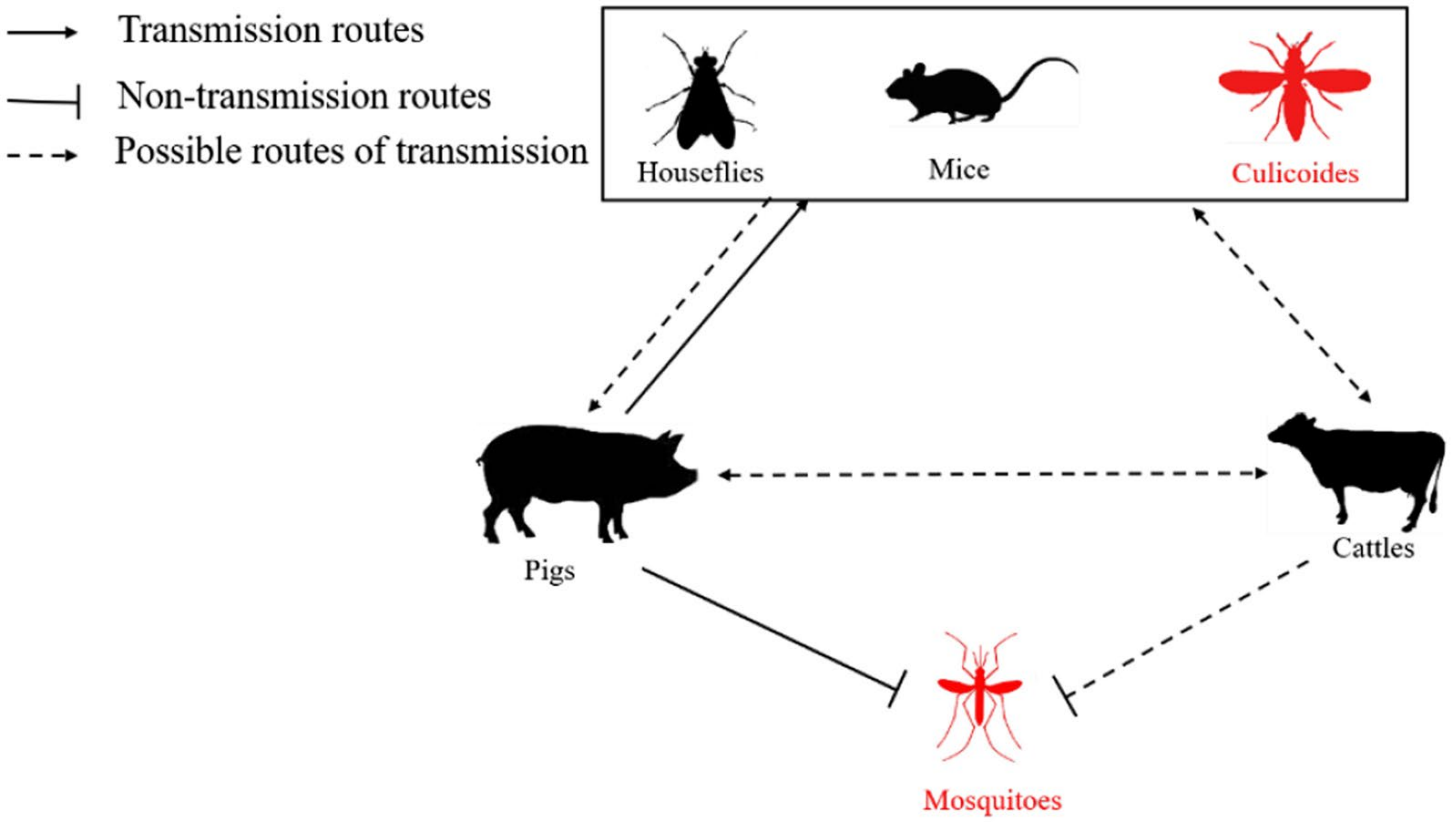
Possible transmission routes of SVV. SVV-positive could be detected in *Culicoides*, but not in mosquitoes in the present study (Red mark in the figure). The solid line indicates the determined transmission route, and the dotted line indicates the speculated propagation route. Mice, *Culicoides*, houseflies and cattles may serve as intermediate transmission hosts of SVV

As previously reported, SVV nucleic acids have been detected in mice and houseflies [23]; and although SVV nucleic acids were not detected in cattle, neutralizing antibodies were present [12], therefore we inferred that cattle may be infected with SVV. So far, the pig is the only host of SVV, therefore we infer that possible transmission routes of SVV, including houseflies, mice, *Culicoides* and cattle may serve as vehicles for SVV transmission and promote the spread of SVV (Fig. 5).

## Discussion

Since the first report of SVV infection in pigs in 2007 [6], porcine infection with this virus has been reported in many countries. The clinical symptoms of porcine SVV are similar to those observed in PIVD, which includes vesicular lesions in the snout, coronary band, and hooves. The loss of piglets within the first 4 days of life if infected with SVV can reach levels of 30–70%; and clinical signs such as muscular weakness, lethargy, excessive salivation, cutaneous hyperemia, neurologic manifestations, and diarrhea can manifest in neonatal pigs [8].

Studies have shown that viremia lasts about 10 days (with viral loads up to about 10^6.5^ copies/ml) and infected individuals present with clinical symptoms for 2–10 days (lethargy, vesicular lesions on the snout and/or feet) when 15-week-old pigs have been inoculated with SVV SD15-26 via the oronasal route in order to study SVV pathogenicity in pigs [24]. 15-week-old pigs have also been inoculated with 10 ml SVV via oral and intranasal routes to study the pathogenicity and adaptive immune responses. The challenged pigs had obvious clinical symptoms (lethargy and lameness, vesicular lesions on the snout and/or feet) from days 4–14, and viremia (approximately 10^8^ copies/ml) lasted for up to 10 days [25]. To compare the pathogenicity of two SVV strains, 90–100 kg pigs challenged with HB-CH-2016 or CH/AH-02/2017 strain via intranasal routes (3 ml and 1.5 ml respectively to each nostril). The results showed that the pathogenicity of the two strains was different, with the pathogenicity of HB-CH-2016 lower than that of CH/AH-02/2017 [18]. To explore whether the pathogenicity of SVV in pigs is related to the age of pigs and infection route, 30–35, 55–65 or 90–100 day old pigs were selected and challenged with SVV-CH-SD via intraoral or intranasal routes and intranasally or intramuscularly, respectively. 90–100 day old pigs inoculated with SVV-CH-SD via intraoral and intranasal, intranasal or intramuscular routes presented with clinical symptoms, while neutralizing antibodies were detected in the other challenge groups [19]. Based on previous studies, the pathogenicity of SVV in pigs may be related to the virulence and the age of pigs, the duration of viremia after challenge is published as about 10–14 days, and some SVV viral strains may cause lesions in some tissues such as the lymph nodes and tonsils. In this review, we studied the pathogenicity of the CH-GX-01-2019 SVV strain in pigs (about 10–15 kg bodyweight) and found that some pigs developed clinical symptoms including blisters when the challenge dose was set at 10 ml. Prior to this, 4 ml or 6 ml SVV (CH-GX-01-2019) was used to cause pig infections but clinical symptoms had not been observed. In this study, we also found that the duration of viremia may be longer than 14 days when pigs are challenged with a high-dose of SVV. It was also discovered that SVV can be distributed in many tissues and that the viral loads in blisters, lymph nodes and tonsils are higher than that in other tissues tested during autopsy on 14 dpi pigs. In addition, a low conceptus age for pigs was selected in this study, which may lay a foundation for low-age animal SVV infection models.

Neutralizing antibodies are believed to play critical roles in promoting resistance to infection. Therefore, it is necessary to explore the production time and duration of neutralizing antibodies following SVV infection in pigs. This may also be helpful for research into SVV pathogenic mechanisms and vaccine development. Previous studies have confirmed that neutralizing antibodies can be detected as early as the 4th day after infection with SVV, and the neutralizing antibody titers reached their highest at about 7–10 dpi [22, 24, 25]. It has also been shown that the neutralizing antibodies in pigs infected with SVV can last for more than 38 days [24]. The titer of neutralizing antibodies produced by different strains of swine infection has differed, but the cross protective effects of neutralizing antibodies among strains suggest that our SVV strains might share the same antigenic epitope [22]. In this manuscript, the neutralizing antibody titers reached their highest levels 10 days after the pigs had been infected with the CH-GX-01-2019 SVV strain, reaching 1:512. The neutralizing antibodies persisted for more than 28 days, and titers did not substantially decay by the 28^th^ day, this may prevent pigs from being reinfected with SVV for a short period of time.

Although pigs are a unique natural host of SVV, studies have confirmed that SVV can be detected in houseflies and mice located on SVV infected pig farms [23]. This may prove that rodents and some insects can promote the spread of SVV. It has also been demonstrated that subclinical symptoms occur following SVV infection in mice, and SVV can also be detected within infected mouse feces within about two weeks; SVV can also be detected in the feces of uninfected mice following exposure to infected mice [12]. These studies provide favorable evidence for the transmission of SVV in rodents. At present, the reports on cattle SVV infection are limited to the detection of neutralizing antibodies in bovine serum, and no SVV virus particles have been detected in RT-PCR experiments, additionally cattle within the study did not show clinical symptoms [12]. We infer that cattle may be the intermediate host of SVV and therefore promote the spread of SVV. In addition, studies have confirmed that SVV has been detected in food, indeed SVV in feed or feed components may risk pig infection for around 37 days [26, 27]. Although several studies have suggested that feed may be potential sources of infection in pigs, the likelihood and severity of this happening in practice may be very low [12]. In this study, we collected mosquitoes and *Culicoides* from pigsties after challenge with SVV. SVV detection within mosquitoes and *Culicoides* was done via RT-PCR, and the results showed that SVV was not detected in the mosquitoes, but was detected in *Culicoides* at a positive rate of 26% (13/50). This result enriches the possible SVV transmission routes and highlights the need for further research into the prevention and control of SVV.

## Conclusions

In conclusion, 5–6 week old pigs infected with SVV (CH-GX-01-2019) developed clinical symptoms such as a low fever and blisters when pigs were challenged with high-doses. Viremia was detected for more than 14 days, and the virus was present in heart, liver, spleen, lung, kidney, submaxillary LN, inguinal LN, intestine, and tongue tissues. The SVV viral load was higher in the tonsils and hooves with blister vesicles, lymph nodes and the tonsils than any of the other tissues. Neutralizing antibodies were detected in pigs at 7–28 dpi, and neutralizing antibody titer levels ranged from 1:128 to 1:512. SVV was detected in *Culicoides* but not in mosquitoes. These studies may help explain the pathogenic mechanism of SVV, enrich the knowledge pertaining to the transmission routes of SVV, and lay the foundation for the future prevention techniques to control the spread of SVV.

## Supplementary Information

**Additional file 1: Table S1**. Primers detection of SVV by qRT-PCR. **Table S2**. Primers detection of SVV.

**Additional file 2: Fig. S1**. SYBR Green I Quantitative Real-time PCR detection method was established. Standard curve (A) and melt curve (B) was established, and the detection threshold is 10^1^ copies/μl.

**Additional file 3: Fig. S2**. Detection of SVV in *Culicoides* samples. 4/10 of the groups were positive, for group 3, 4, 5, 6 (A); the results of separate sample detection showed that sample 13, 14, 15, 16, 17, 18, 19, 20, 21, 22, 23, 25, 30 was positive (B). **Table S3**. Results of SVV were detected in mosquitoes and *Culicoides*.

## Data Availability

All data generated or analyzed during this study are included in this published article.

## Abbreviations

SVV: Seneca Valley virus
dpi: Days post infection
FMDV: Food-and-mouth disease virus
VSV: Vesicular stomatitis virus
SVDV: Swine vesicular disease virus
VESV: Vesicular exanthema of swine virus
BHK-21: Baby hamster kidney cell line
DMEM: Modified Eagle’s medium
FBS: Fetal bovine serum
RT-PCR: Reverse transcription polymerase chain reaction
qRT-PCR: Quantitative real-time PCR
LN: Lymph nodes
VNT: Virus neutralizing antibody test
TCID50/ml: 50% Tissue culture infective doses per milliliter
CPE: Cytopathic effect
IFA: Indirect immunofluorescence assay
PIVD: Porcine idiopathic vesicular disease
