# Supplementary material for: Pathogenicity of Seneca Valley virus in pigs and detection in Culicoides from an infected pig farm
Source: Virol J. 2021 Oct 21;18:209. doi: 10.1186/s12985-021-01679-w (PMC8529370; doi:10.1186/s12985-021-01679-w)
Supplement: Supplementary file 1 — Additional file 1: Table S1. Primers detection of SVV by qRT-PCR. Table S2. Primers detection of SVV. [file 12985_2021_1679_MOESM1_ESM.docx]

Additional file 1: Table S1 Primers detection of SVV by qRT-PCR

| Name of Primers | Primers (5’→3’) | Size of Products (bp) |
| --- | --- | --- |
| SVV-qPCR-F | CAGCAAGGGTGGAAAGGTA | 159 |
| SVV-qPCR-R | TTAGGACGGGGGATAAAGG |  |

Additional file 1: Table S2 Primers detection of SVV

| Name of Primers | Primers (5’→3’) | Size of Products (bp) |
| --- | --- | --- |
| SVV-F | ACTTTTGTCTTCTAACACCCCG | 581 |
| SVV-R | TTGCATCAGCATCTTCTGCTT |  |
