# Supplementary material for: Pathogenicity of Seneca Valley virus in pigs and detection in Culicoides from an infected pig farm
Source: Virol J. 2021 Oct 21;18:209. doi: 10.1186/s12985-021-01679-w (PMC8529370; doi:10.1186/s12985-021-01679-w)
Supplement: Supplementary file 2 — Additional file 2: Fig. S1. SYBR Green I Quantitative Real-time PCR detection method was established. Standard curve (A) and melt curve (B) was established, and the detection threshold is 101 copies/μl. [file 12985_2021_1679_MOESM2_ESM.docx]

**Additional file 2: Fig. S1**


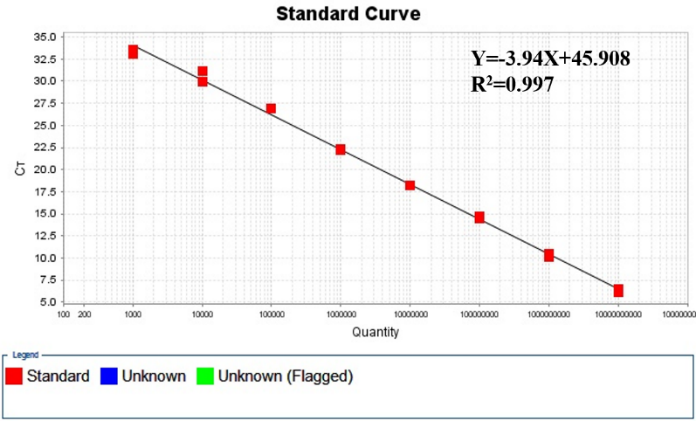
(A)


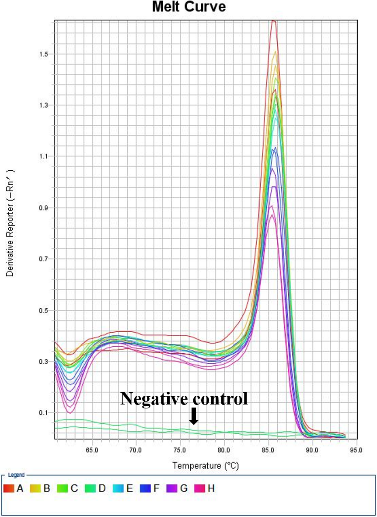
(B)

**Additional file 2: Fig. S1 SYBR Green I Quantitative Real-time PCR detection method was established.** Standard curve (A) and melt curve (B) was established, and the detection threshold is 10^1^ copies/μl.
