# Supplementary material for: Pathogenicity of Seneca Valley virus in pigs and detection in Culicoides from an infected pig farm
Source: Virol J. 2021 Oct 21;18:209. doi: 10.1186/s12985-021-01679-w (PMC8529370; doi:10.1186/s12985-021-01679-w)
Supplement: Supplementary file 3 — Additional file 3: Fig. S2. Detection of SVV in Culicoides samples. 4/10 of the groups were positive, for group 3, 4, 5, 6 (A); the results of separate sample detection showed that sample 13, 14, 15, 16, 17, 18, 19, 20, 21, 22, 23, 25, 30 was positive (B). Table S3. Results of SVV were detected in mosquitoes and Culicoides. [file 12985_2021_1679_MOESM3_ESM.docx]

**Additional file 3: Fig. S2**


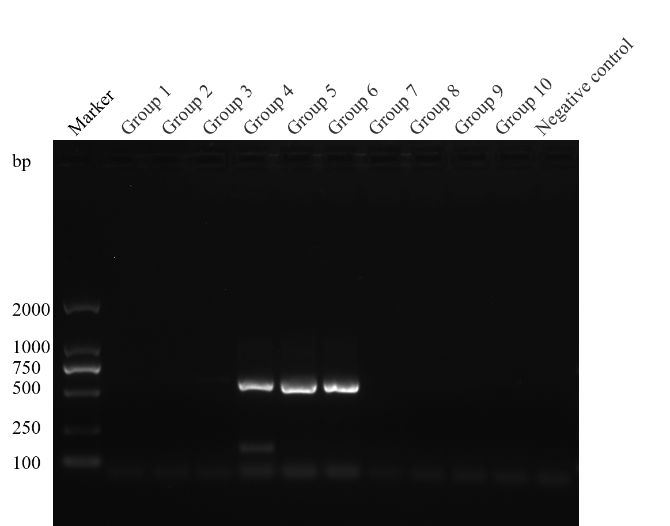
（A）

（B）


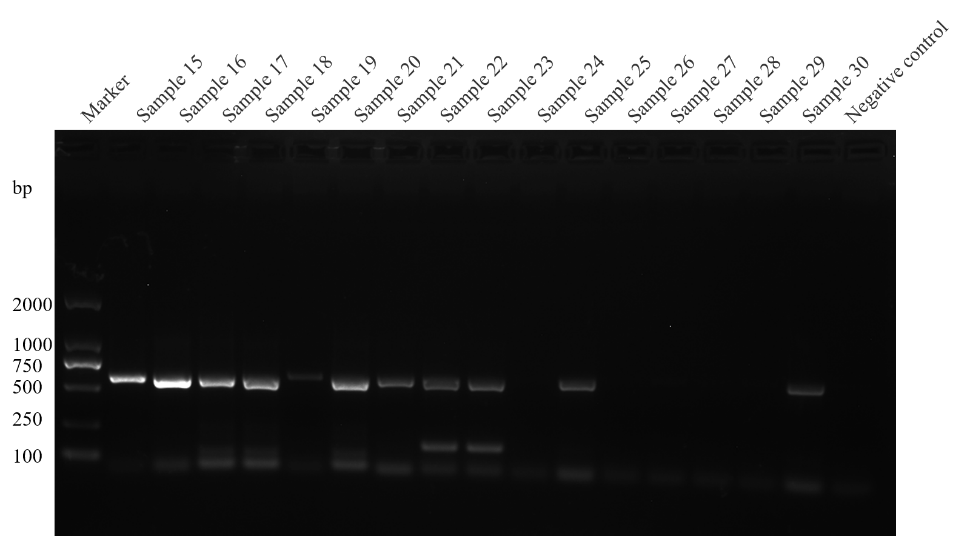

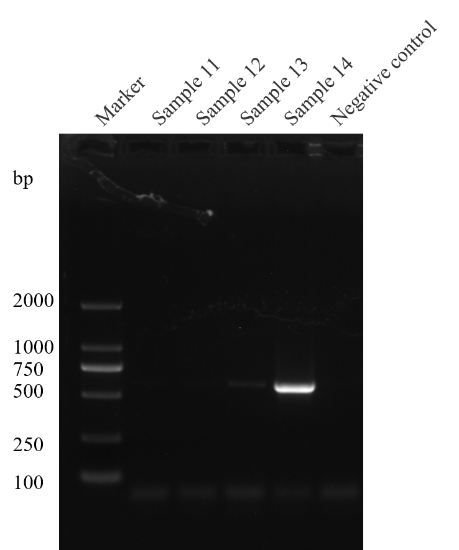


**Additional file 3: Fig. S2 Detection of SVV in *Culicoides* samples.** 4/10 of the groups were positive, for group 3, 4, 5, 6 (A); the results of separate sample detection showed that sample 13, 14, 15, 16, 17, 18, 19, 20, 21, 22, 23, 25, 30 was positive (B).

Additional file 3: Table S3 Results of SVV were detected in mosquitoes and *Culicoides*

| Species | Groups | Samples | PCR results | Positive sample |
| --- | --- | --- | --- | --- |
| Mosquitoes | Group 1 |  | Negative |  |
|  | Group 2 |  | Negative |  |
|  | Group 3 |  | Negative |  |
|  | Group 4 |  | Negative |  |
|  | Group 5 |  | Negative |  |
|  | Group 6 |  | Negative |  |
|  | Group 7 |  | Negative |  |
|  | Group 8 |  | Negative |  |
|  | Group 9 |  | Negative |  |
|  | Group 10 |  | Negative |  |
| Culicoides | Group 1 | 1-5 | Negative |  |
|  | Group 2 | 6-10 | Negative |  |
|  | Group 3 | 11-15 | Positive | 13, 14, 15 |
|  | Group 4 | 16-20 | Positive | 16, 17, 18, 19, 20 |
|  | Group 5 | 21-25 | Positive | 21, 22, 23, 25 |
|  | Group 6 | 26-30 | Positive | 30 |
|  | Group 7 | 31-35 | Negative |  |
|  | Group 8 | 36-40 | Negative |  |
|  | Group 9 | 41-45 | Negative |  |
|  | Group 10 | 46-50 | Negative |  |
